# Association between hematocrit in the first two hours of life and retinopathy during prematurity: a retrospective study from DRYAD

**DOI:** 10.1186/s12887-025-05533-8

**Published:** 2025-03-08

**Authors:** Xiaohui Kong, Huabin Wang, Ru Yang, Min Zhang, Chengshuai Li, Rui Zhang, Lihua Wei, Jing Xu, Xueyun Ren

**Affiliations:** 1 Department of Pediatrics, Affiliated Hospital of Jining Medical University, Jining Medical University, Jining, China; 2 Shandong Provincial Key Medical and Health Discipline of Pediatric Internal Medicine, Affiliated Hospital of Jining Medical University), Jining, China; 3 Jining Key Laboratory for Prevention and Treatment of Severe Infections in Children, Jining, China

**Keywords:** Hematocrit, Retinopathy of prematurity, Premature infant, Retrospective study

## Abstract

**Introduction:**

Our study investigated the association between hematocrit in the first two hours (HCT2h) of life and retinopathy of prematurity (ROP).

**Methods:**

Data were obtained from an observational study of the DRYAD database. The study was conducted at the Santa Clara Valley Medical Center (SCVMC). Data on preterm babies whose gestational age (GA) was < 34 weeks were collected prospectively from January 2008 to February 2014. Logistic regression was applied to explore the association between HCT2h and ROP.

**Results:**

A total of 326 very preterm infants born at or earlier than 34 weeks were included. The incidence of any ROP was 23.9%, and the incidence of severe ROP was 4.6%. The HCT2h, birth weight, GA, Apgar1 min, and Apgar5 min of any ROP group were significantly lower than those of preterm babies without ROP (*p* < 0.001). Sex differences, the rate of multiples, and delivery mode between the two groups were not statistically significant (*p* > 0.05). We classified HCT2h into three levels, and after multivariate logistic regression, we found that high HCT2h remained a significant protective factor against ROP (*p* < 0.001). Through subgroup analysis, we observed that among preterm infants with a GA of 28 weeks or more, there was a significant inverse association between a 1% increase in HCT2h and a 17% reduction in the occurrence of ROP.

**Conclusion:**

We found that HCT2h may be an effective biomarker for identifying the risk of ROP of very preterm infants born between 28 and 34 weeks of gestation.

**Trial registration:**

This was a retrospective study and the data were from the DRYAD database. Santa Clara Valley Medical Center’s (SCVMC) ethical committee reviewed and approved the studies involving human participants. Informed consent was waived for this study. We did not perform any extra interventions.

**Supplementary Information:**

The online version contains supplementary material available at 10.1186/s12887-025-05533-8.

## Introduction

Retinopathy of prematurity (ROP) is a neovascular disorder affecting the immature retina. The prevalence and severity of retinopathy of prematurity (ROP) vary considerably between different countries. A cohort study demonstrated that ROP affects approximately 26.5–43% of infants in the United States, with a higher incidence observed in low- and middle-income countries [1]. Because of the lack of effective and efficient screening, it is the primary cause of preventable visual impairment worldwide, especially in developing countries [2, 3]. Screening at-risk preterm infants following birth in the neonatal intensive care unit (NICU) is imperative for ROP management, as early detection and treatment of severe disease can decrease the risk of blindness.

Numerous studies have explored risk factors for ROP to identify a way to screen for ROP early. Several perinatal [4] and postnatal factors, such as elevated maternal leukocyte count and clinical chorioamnionitis, have been associated with ROP, and the most common and well-studied perinatal and neonatal risk factors for the development of severe ROP are hyperoxemia, hypoxia, and prolonged respiratory support [1]. The current screening criteria for ROP, birth weight (BW) and gestational age (GA), have high sensitivity but low specificity [5], and others use postnatal weight gain or insulin-like growth factor 1 (IGF-1) levels to screen for ROP. Ana C. Almeida investigated the associations between hyperglycemia, glycated albumin (GlyA), and ROP. The study revealed that hyperglycemia, but not GlyA, was a significant risk factor for ROP, outweighing other recognized risk factors [6]. Some studies have examined the effect of cord blood magnesium levels on ROP and found no relationship [7]. Low platelet counts and serum VEGF-A levels have been found to be associated with ROP development [8]. Moreover, another study showed that oral supplementation with vitamin E and DHA may decrease oxidative damage markers and effectively prevent ROP development in VLBW infants with RDS [9, 10]. Very early postnatal biomarkers predictive of ROP outcome have not yet been identified.

Previous studies have indicated that the minimization of anemia may prove beneficial in the reduction of ROP rates [2]. Hematocrit indicates the degree of anemia and, indirectly, of tissue oxygenation in preterm infants. Does the hematocrit level in the early life of preterm infants correlate with the risk of ROP? We conducted a secondary data analysis based on open-source data from a previously published paper [11]. The objective of this study was to explore the association between HCT2h and ROP among very preterm infants born at or earlier than 34 weeks.

## Methods

### Data source

Detailed citations were used to obtain information on the studies of Song Dongli, Jegatheesan Priya, DeSandre Glenn, and Govindaswami Balaji [11]. All data files are available from the data dryad database (doi: 10.5061/dryad.4q3d3).

### Study population

The original study was a prospective observational study conducted from January 2008 to April 2014 at the Santa Clara Valley Medical Center (SCVMC). The Ethical Committee of the Affiliated Hospital of Jining Medical University approved the study (2024C134), and the informed consent was waived.

Following the current + screening guidelines, all infants with a GA < 34 weeks who underwent delayed cord clamping (DCC) (30–75 s) and whose hematocrits were < 2 h were included. Infants without hematocrits within 2 h of life were excluded.

### Data collection and measurements

The following variables of the enrolled participants in the database were included: GA, BW, sex, Ans [antenatal steroid (any and > 48-hour exposure)], delivery mode, multiple pregnancy status, Apgar status at 1 and 5 min, Etippv (delivery room intubation and positive pressure ventilation), Drccompmeds (delivery room chest compression and/or resuscitation medication), Atemp (temperature at NICU admission), surfactant administration, Intub1d (intubation within the first 24 h of life), any intubation and any transfusion (intubation and red cell transfusion at any time during the NICU stay), and Pkbili (peak bilirubin). Hematocrits included in this analysis were those collected at < 2 h, and those obtained after blood transfusion were excluded. The neonatal morbidities were as follows: IVH, late-onset sepsis (LOS), NEC, chronic lung disease (CLD), any ROP defined as stage 1–3, and severe ROP defined as > stage 2 or with plus disease or requiring surgery or anti-vascular endothelial growth factor treatment for ROP.

### Statistical analysis

The R software program (http://www.R-project.org, The R Foundation) and Free Statistics software version 1.9 were used for all the statistical studies. *P* < 0.05 indicated statistical significance. The distribution of the study participants’ baseline values for each of the several HCT2h categories (tertiles) is displayed. Percentages (%) are used to express categorical variables. Normally distributed continuous variables are given as the mean and standard deviation (SD) or, if skewed, as the median and interquartile range (IQR). The categorical variables were compared with regularly distributed and nonnormally distributed continuous variables using Q-way analysis of variance (normal distribution), the Kruskal‒Wallis test (skewed distribution), and the chi‒squared test (categorical variables).

This study employed multivariable logistic regression analysis to evaluate the potential independent relationship between HCT2h and the occurrence of ROP. We adjusted for features that altered the adjusted odds ratio (OR) by at least 10% when they were included in this model. The mode of delivery, sex, BW, and GA were all periodically adjusted. The assessment of collinearity was performed before multivariate analyses. Following the guidelines in the statement “Strengthening the Reporting of Observational Studies in Epidemiology,” HCT2h was examined as a continuous variable in each of the modified models [3]. At the same time, we displayed the outcomes of the following analyses: unadjusted, minimally adjusted (BW, GA, sex, delivery mode), multiply adjusted (BW, GA, sex, delivery mode, Multiple Pregnancy, Ans, Apgar1, Apgar5), and fully adjusted (BW, GA, sex, delivery mode, Multiple Pregnancy, Ans, Apgar1, Apgar5, Etippv, Drccompmeds, Atemp, Surfactant, Pneumothorax, Intub1d, Anytransfusion, Pkbili).

Stratified regression models were used for subgroup analysis. Likelihood ratio tests were used to examine the changes and interactions among subgroups.

## Results

### Baseline characteristics

The DRYAD data of 352 extremely preterm babies were retrieved. A total of 326 preterm individuals whose hematocrits were collected within the first two hours of life were analyzed, and 26 preterm patients without HCT2h data were removed from the analysis. Of the 326 infants, 78 (23.9%) had ROP, and the comparison of clinical parameters between the no ROP and ROP groups was shown in Table 1. The baseline characteristics of the selected participants according to tertiles of HCT2h are shown in Table 2. The patients with elevated HCT2h levels exhibited enhanced BW, GA, and Apgar scores at the 1st and 5th minutes post-partum, along with elevated peak bilirubin levels. They demonstrated a reduced incidence of pulmonary surfactant administration and intubation within the NICU. The mean BW and GA were 1375.5 ± 461.4 g and 29.6 ± 2.6 w, respectively, and there were 122 males (37.4%) and 204 females (62.6%). A total of 265 (81.3%) singleton pregnancies and 61 (18.7%) multiple pregnancies were recorded. Only nine (2.8%) preterm newborns did not receive glucocorticoids prior to delivery, out of the 317 (97.2%) who received prenatal steroids. Of the deliveries, 196 were by cesarean Sect. (60%), and 130 were by normal delivery (39.9%). The Apgar score at one minute was 5.9 ± 2.3, and at five minutes, it was 7.5 ± 1.7. 96% of patients received resuscitation medicine and/or chest compression in the delivery room, while 82.5% received intubation and positive pressure ventilation. Her temperature at admission was 36.9 ± 0.5 °C. Among the preterm newborns, 260 (79.8%) received lung surfactant, whereas 66 (20.2%) did not. Within 24 h of birth, 91 (27.9%) preterm infants were extubated, while 111 (34%) accepted intubation while they were in the NICU. Pneumothorax occurred in eleven (3.4%) preterm neonates, and 92 (28.2%) received blood transfusions. All of these preterm babies had a peak bilirubin concentration of 8.4 ± 2.6 mg/dL.

**Table 1:** Comparison of clinical parameters between the no ROP and ROP groups

Variables	Total (*n* = 326)	No ROP (*n* = 248)	Any ROP (*n* = 78)	*p* -value
HCT2(%)	48.3 ± 7.0	49.9 ± 6.5	43.1 ± 6.0	< 0.001
BW (g)	1375.5 ± 461.4	1510.7 ± 418.9	945.8 ± 299.8	< 0.001
GA(w)	29.6 ± 2.6	30.4 ± 2.1	27.0 ± 2.0	< 0.001
Sex				0.627
female	122 (37.4)	91 (36.7)	31 (39.7)	
male	204 (62.6)	157 (63.3)	47 (60.3)	
Multiples				0.595
No	265 (81.3)	200 (80.6)	65 (83.3)	
Yes	61 (18.7)	48 (19.4)	13 (16.7)	
Delivery Mode				0.176
Cesarean section	196 (60.1)	144 (58.1)	52 (66.7)	
Vaginal delivery	130 (39.9)	104 (41.9)	26 (33.3)	
Apgar1	5.9 ± 2.3	6.2 ± 2.2	5.0 ± 2.4	< 0.001
Apgar5	7.5 ± 1.7	7.8 ± 1.6	6.7 ± 1.8	< 0.001

HCT2, hematocrit in the first two hours of life; BW, birth weight; GA, gestational age; Multiple, multiple pregnancy; Apgar1,1-minute Apgar; Apgar5,5-minute Apgar

**Table 2:** Clinical characteristics of the study population according to HCT2 (hematocrit in the first two hours of life)

Variables	Total (*n* = 326)	Q1 (*n* = 108)	Q2 (*n* = 109)	Q3 (*n* = 109)	*p* -value
BW(g)	1375.5 ± 461.4	1141.0 ± 434.8	1397.7 ± 471.0	1585.7 ± 362.6	< 0.001
GA(w)	29.6 ± 2.6	28.0 ± 2.6	29.7 ± 2.4	31.1 ± 1.6	< 0.001
Sex					0.254
female	122 (37.4)	47 (43.5)	36 (33)	39 (35.8)	
male	204 (62.6)	61 (56.5)	73 (67)	70 (64.2)	
Multiple Pregnancy					0.881
No	265 (81.3)	89 (82.4)	89 (81.7)	87 (79.8)	
Yes	61 (18.7)	19 (17.6)	20 (18.3)	22 (20.2)	
Ans					1
No	9 (2.8)	3 (2.8)	3 (2.8)	3 (2.8)	
Yes	317 (97.2)	105 (97.2)	106 (97.2)	106 (97.2)	
Delivery Mode					0.413
Cesarean section	196 (60.1)	68 (63)	60 (55)	68 (62.4)	
Vaginal delivery	130 (39.9)	40 (37)	49 (45)	41 (37.6)	
Apgar1	5.9 ± 2.3	5.0 ± 2.3	6.1 ± 2.2	6.7 ± 1.9	< 0.001
Apgar5	7.5 ± 1.7	7.0 ± 1.7	7.5 ± 1.9	8.1 ± 1.4	< 0.001
Etippv					< 0.001
No	269 (82.5)	67 (62)	97 (89)	105 (96.3)	
Yes	57 (17.5)	41 (38)	12 (11)	4 (3.7)	
Drccompmeds					0.045
No	313 (96.0)	100 (92.6)	105 (96.3)	108 (99.1)	
Yes	13 (4.0)	8 (7.4)	4 (3.7)	1 (0.9)	
Atemp(℃)	36.9 ± 0.5	37.0 ± 0.5	36.9 ± 0.6	36.9 ± 0.4	0.348
Surfactant					< 0.001
No	260 (79.8)	65 (60.2)	92 (84.4)	103 (94.5)	
Yes	66 (20.2)	43 (39.8)	17 (15.6)	6 (5.5)	
Intub1d					< 0.001
No	235 (72.1)	51 (47.2)	86 (78.9)	98 (89.9)	
Yes	91 (27.9)	57 (52.8)	23 (21.1)	11 (10.1)	
Pneumothorax					0.224
No	315 (96.6)	103 (95.4)	104 (95.4)	108 (99.1)	
Yes	11 (3.4)	5 (4.6)	5 (4.6)	1 (0.9)	
Any intub					< 0.001
No	215 (66.0)	43 (39.8)	77 (70.6)	95 (87.2)	
Yes	111 (34.0)	65 (60.2)	32 (29.4)	14 (12.8)	
Any transfusion					< 0.001
No	234 (71.8)	48 (44.4)	82 (75.2)	104 (95.4)	
Yes	92 (28.2)	60 (55.6)	27 (24.8)	5 (4.6)	
Pkbili(mg/dl)	8.4 ± 2.6	7.1 ± 2.5	8.6 ± 2.6	9.6 ± 2.1	< 0.001
Anyrop					< 0.001
No	248 (76.1)	58 (53.7)	87 (79.8)	103 (94.5)	
Yes	78 (23.9)	50 (46.3)	22 (20.2)	6 (5.5)	

The data are presented as the means ± SDs or numbers (proportions) for categorical variables

BW, birth weight; GA, gestational age; Ans, antenatal steroids; Apgar1,1-minute Apgar; Apgar5,5-minute Apgar; Etippv, delivery room intubation and positive pressure ventilation; Drccompmeds, delivery room chest compression and/or resuscitation medication; Atemp, admission temperature in Celsius; Intub1d, intubation within 24 h of life; Any Intub, intubation any time during the NICU stay; Any transfusion, any pRBC transfusion during the NICU stay; Pkbili, peak bilirubin; Anyrop, retinopathy of prematurity (stage 1–3); Q1, Q2, and Q3 are quartiles of the HCT2 (hematocrit in the first two hours of life)

### Univariate and multivariate analyses of HCT2h and ROP

The results of the univariate logistic regression model analyses are shown in Table 3. Risk factors for ROP are Etippv, positive pressure ventilation by endotracheal intubation in the delivery room, Drccompmeds, delivery room chest compression and/or resuscitation medication, surfactant, pneumothorax, intubation within 24 h of life, and intubation any time during the NICU stay. The protective factors included gestational age, 1-minute Apgar score, 5-minute Apgar score, admission temperature in Celsius, peak bilirubin, HCT2h, and vaginal delivery. Table 4 displays multivariate logistic regression models. HCT2h was negatively linked with the incidence of ROP in all models, including the completely adjusted model (adjusted for all the covariates as listed in Table 3) and the nonadjusted, minimally adjusted, and multiply adjusted models. One increase in HCT2h was linked, after multiple corrections, to a 9% reduction in the incidence of ROP [OR = 0.91; 95% confidence interval (CI): 0.84,0.97; *p* = 0.008; Table 4] in model 3. For the sensitivity analysis, we also considered HCT2h as a categorical variable (tertile), and we found a similar tendency ( *p-value* for the trend was 0.004; Table 4).

**Table 3:** Univariate logistic regression models evaluating the association between HCT2 (hematocrit in the first two hours of life) and retinopathy of prematurity

Variable	OR_95CI	p_value
BW	1 (0.99 ~ 1)	< 0.001
GA	0.47 (0.39 ~ 0.55)	< 0.001
Sex2	0.88 (0.52 ~ 1.49)	0.637
Multiple Pregnancy	0.77 (0.39 ~ 1.52)	0.459
Ans 1	0.24 (0.05 ~ 1.1)	0.066
Apgar1	0.73 (0.65 ~ 0.83)	< 0.001
Apgar5	0.64 (0.54 ~ 0.75)	< 0.001
Etippv	9.12 (4.56 ~ 18.27)	< 0.001
Drccompmeds	4.79 (1.32 ~ 17.45)	0.018
Atemp	0.85 (0.51 ~ 1.39)	0.512
Surfactant	6.76 (3.65 ~ 12.51)	< 0.001
Pneumothorax	9.67 (1.91 ~ 48.95)	0.006
Intub1d	6.99 (3.94 ~ 12.39)	< 0.001
Anyintub	9.15 (5.14 ~ 16.31)	< 0.001
Any transfusion	14.99 (8.11 ~ 27.7)	< 0.001
Pkbili	0.56 (0.48 ~ 0.65)	< 0.001
HCT2	0.83 (0.79 ~ 0.88)	< 0.001
Vaginal delivery	0.68 (0.4 ~ 1.17)	0.163
Hypothermia1	1.13 (0.29 ~ 4.37)	0.859

BW, birth weight; GA, gestational age; Ans, antenatal steroids; Apgar1,1-minute Ap Drccompmeds, delivery room chest compression and/or resuscitation medication gar; Apgar5,5-minute Apgar; Etippv, delivery room intubation and positive pressure ventilation; Drccompmeds, delivery room chest compression and/or resuscitation medication; Atemp, admission temperature in Celsius; Intub1d, intubation within 24 h of life; Any Intub, intubation any time during the NICU stay; Any transfusion, any pRBC transfusion during the NICU stay; Pkbili, peak bilirubin

**Table 4:** Multivariable-adjusted ORs and 95% CIs of the HCT2 index quartiles associated with retinopathy of prematurity

Variable	Unadjusted		Model1		Model2		Model3	
	OR(95%CI)	*p* value	OR(95%CI)	*p* value	OR(95%CI)	*p* value	OR(95%CI)	*p* value
HCT2	0.83 (0.79 ~ 0.88)	< 0.001	0.91 (0.86 ~ 0.97)	0.004	0.9 (0.84 ~ 0.96)	0.003	0.91 (0.84 ~ 0.97)	0.008
HCT tertiles								
Q1	Ref		Ref		Ref		Ref	
Q2	0.27 (0.14 ~ 0.49)	< 0.001	0.45 (0.2 ~ 1.03)	0.057	0.47 (0.2 ~ 1.09)	0.08	0.46 (0.18 ~ 1.15)	0.096
Q3	0.06 (0.02 ~ 0.15)	< 0.001	0.22 (0.08 ~ 0.66)	0.007	0.2 (0.07 ~ 0.63)	0.006	0.23 (0.07 ~ 0.78)	0.018
p for trend	0.25 (0.17 ~ 0.37)	< 0.001	0.47 (0.28 ~ 0.79)	0.004	0.46 (0.27 ~ 0.78)	0.004	0.48 (0.27 ~ 0.86)	0.013

Model 1 adjusts for BW, GA, sex, and delivery mode

Model 2 was adjusted for Model 1 + Multiple Pregnancy + Ans + Apgar1 + Apgar5

Model 3 is adjusted for Model 1 + Model 2 + Etippv + Drccompmeds + Atemp + Surfactant + Pneumothorax + Intub1d + Anytransfusion + Pkbili

Ref, reference; HCT2, hematocrit in the first two hours of life; BW, birth weight; GA, gestational age; Ans, antenatal steroids; Apgar1,1-minute Apgar; Apgar5,5-minute Apgar; Etippv, delivery room intubation and positive pressure ventilation; Drccompmeds, delivery room chest compression and/or resuscitation medication; Atemp, admission temperature in Celsius; Intub1d, intubation within 24 h of life; Any Intub, intubation any time during the NICU stay; Any transfusion, any pRBC transfusion during the NICU stay; Pkbili, peak bilirubin. Q1, Q2 and Q3 are quartiles of HCT2 (hematocrit in the first 2 h of life)

### Subgroup analyses by adjusted potential effect confounders

**Fig. 1:**
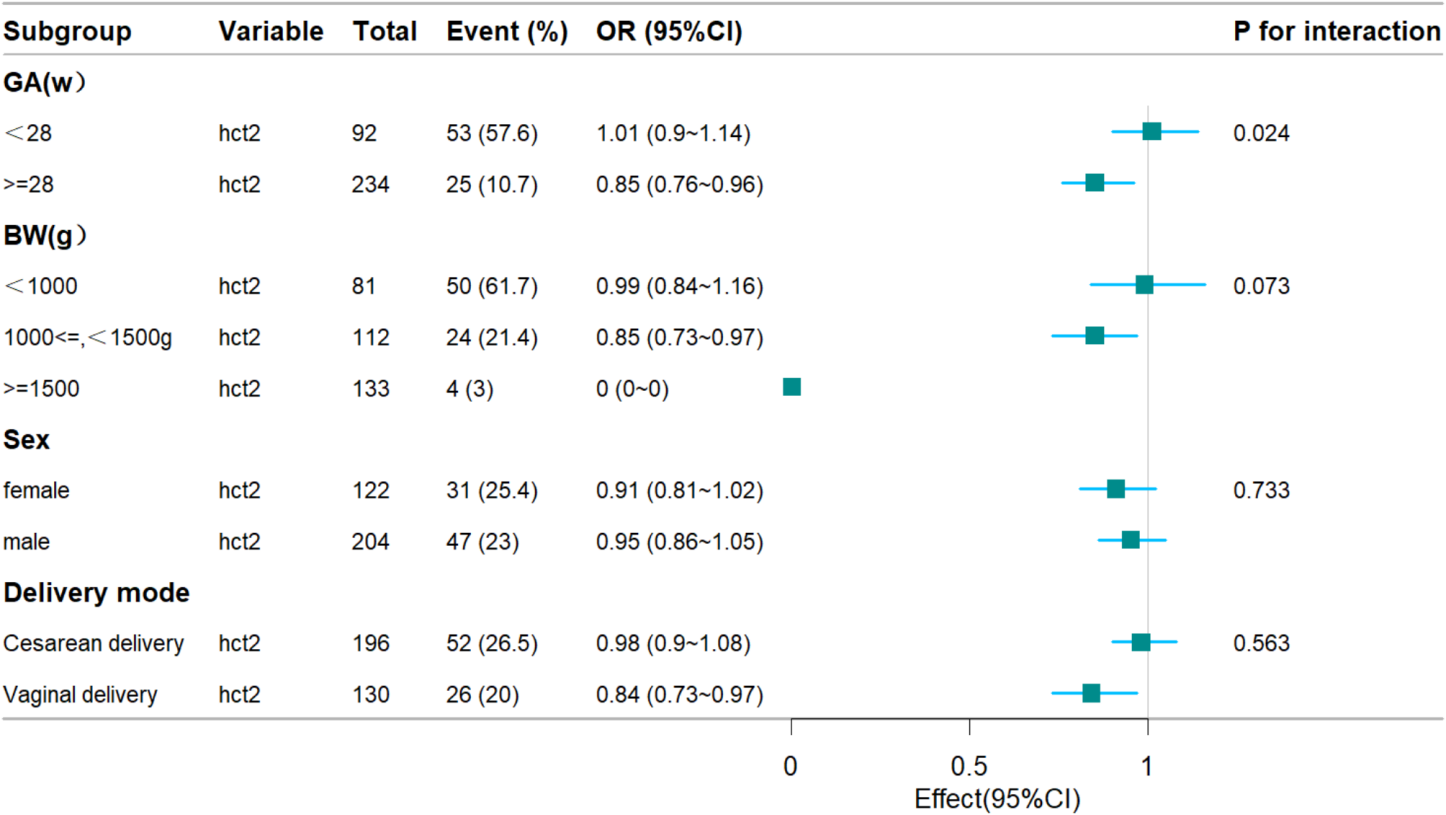
Forest plot of the association between HCT2 and any ROP

Table 5; Fig. 1 show that the interactions for BW, sex, and delivery mode in the subgroup analysis were not statistically significant. In the subgroups, we found that GA interacted with HCT2 (*p* < 0.05).

**Table 5:** Subgroup analyses of HCT2 and ROP patients

Subgroup	Variable	*n*.total	*n*.event_%	crude.OR_95CI	crude.P_value		adj.OR_95CI	adj.p_value	*p*.for.interaction
**GA(w)**									
<28	HCT2	92	53 (57.6)	0.95 (0.88 ~ 1.03)		0.188	1.01 (0.9 ~ 1.14)	0.819	0.024
≥ 28	HCT2	234	25 (10.7)	0.81 (0.75 ~ 0.88)		< 0.001	0.85 (0.76 ~ 0.96)	0.006	
**BW(g)**									
<1000	HCT2	81	50 (61.7)	0.93 (0.85 ~ 1.02)		0.124	0.99 (0.84 ~ 1.16)	0.892	0.073
≥ 1000,<1500	HCT2	112	24 (21.4)	0.87 (0.8 ~ 0.95)		0.001	0.85 (0.73 ~ 0.97)	0.02	
≥ 1500	HCT2	133	4 (3)	0.78 (0.66 ~ 0.93)		0.006	0 (0 ~ 0)	< 0.001	
**Sex**									
female	HCT2	122	31 (25.4)	0.84 (0.77 ~ 0.91)		< 0.001	0.91 (0.81 ~ 1.02)	0.114	0.733
male	HCT2	204	47 (23)	0.83 (0.77 ~ 0.89)		< 0.001	0.95 (0.86 ~ 1.05)	0.283	
**Delivery Mode**									
Cesarean section	HCT2	196	52 (26.5)	0.85 (0.8 ~ 0.9)		< 0.001	0.98 (0.9 ~ 1.08)	0.718	0.563
Vaginal delivery	HCT2	130	26 (20)	0.8 (0.72 ~ 0.88)		< 0.001	0.84 (0.73 ~ 0.97)	0.018	

## Discussion

The development of ROP is mainly attributed to prematurity and oxygen delivery. Although the pathophysiology of ROP has been widely investigated, it remains somewhat unknown. There are apparently common risk factors, such as low GA and low BW. The univariate logistic regression models of our study indicated that BW, GA, Apgar1, Apgre5, delivery room intubation and positive pressure ventilation, delivery room chest compressions and/or resuscitation medications, surfactant, pneumothorax, intubation within 24 h of life, intubation any time during the NICU stay, any pRBC transfusion during the NICU stay, and peak bilirubin were all risk factors for ROP development. Our data also showed that premature infants with ROP had significantly lower GA and BW and lower Apgar1 and Apgar5 than non-ROP infants, which was consistent with the findings of previous studies [12].

Determining a possible biomarker of ROP progression in humans is challenging. In recent years, several authors have investigated the possible relationships between routinely tested blood parameters and ROP development, such as a reduced platelet count, and thrombocytopenia has been correlated with severe ROP outcomes [13]. Our study explored the correlation between the incidence of ROP and HCT2h in extremely preterm infants born before or at 34 weeks of pregnancy. In our study, HCT2h was the earliest blood test parameter that can avoid confounding factors, such as systemic treatment, and is easy to obtain. The incidence of ROP and HCT2h were negatively correlated according to the multiple adjustment model. There was an approximately linear correlation between HCT2h and the incidence of ROP when we looked at HCT2h as a categorical variable. This may be related to the decrease in the oxygen-carrying capacity of the blood caused by low HCT and low hemoglobin [14, 15].

The results suggest that including early HCT among routinely measured blood parameters may help to identify preterm infants with GA at 28–34 weeks who may be more prone to ROP well in advance. In this way, we can screen preterm infants at high risk of ROP as early as possible and provide active treatment, thereby reducing the incidence of ROP. The aforementioned statement provides us with novel concepts for the early detection of ROP and for mitigating the risk of ROP through proactive intervention. However, does that mean that if the HCT2 of preterm babies is low, we should administer a blood transfusion. A multicenter randomized trial showed that umbilical cord red blood cell (RBC) transfusion may reduce the severity of ROP in neonates of extreme gestational age [16]. Moreover, another prospective study compared liberal and restrictive RBC transfusions and demonstrated that no statistically significant differences were observed in ROP incidence between these two groups [17, 18]. However, another study has shown that multiple transfusions of RBCs and PLTs are associated with a higher stage of ROP development [19]. Previous studies have investigated the potential impact of erythropoietin (EPO) supplementation, iron, or blood transfusions on the development of ROP [20–22], but the results have been inconsistent. Repeated transfusions in preterm infants with anemia of prematurity to replace fetal hemoglobin (HbF) with adult hemoglobin (HbA) are associated with a greater incidence of ROP [23, 24]. Umbilical cord blood (UCB) contains growth factors and progenitor cells that may affect ROP. Autologous UCB transfusions could reduce the risks associated with heterologous blood products and offer benefits by maintaining physiological HbF levels and potentially improving postnatal development [25]. DCC may be safer or placental transfusion may be performed for preterm babies with a GA between 28 and 32 weeks. However, for extremely preterm infants younger than 28 weeks, DCC or UCB infusion cannot reduce the incidence of ROP, which may be related to the extremely immature development of these preterm infants.

The role of HCT2 in ROP is not completely understood. The correlation between low HCT2 and ROP development suggests that blood tests after birth may be predictive of an unfavorable course of ROP and may help to elucidate the pathophysiology of ROP. Early identification of ‘at-risk’ preterm babies through a simple blood test would also help to identify those babies who are more likely to develop ROP and who need to be monitored more closely, ideally improving the management of this potentially blinding condition. Future multicenter, prospective clinical studies and basic research are required to determine the deeper relationship between low HCT2 and ROP and whether preterm infants with low HCT should receive active transfusion.

There were certain limitations to this study as well. First, the small sample size of 326 extremely preterm newborns in this study warrants larger sample sizes in future multicenter studies. The present study included only preterm infants who underwent DCC. It would be beneficial to include more premature infants with or without DCC in future research. Second, this study included only American patients. HCT2h is not frequently evaluated in extremely preterm infants in China. When extrapolating the findings of this study to other populations, caution should be exercised. Third, some eligible patients may have been removed from the initial study because this was a secondary analysis of data from a previously published study.

## Conclusions

HCT2h may be an effective biomarker for identifying very preterm infants born at or before 34 weeks of gestation at risk for ROP and allow earlier intervention to improve the prognosis of premature infants. Further research on this correlation is warranted.

## Electronic supplementary material

Below is the link to the electronic supplementary material.

Supplementary Material 1

## Data Availability

The original contributions presented in the study are included in the article/Supplementary Material. Further inquiries can be directed to the corresponding author.
