# Supplementary material for: Association between hematocrit in the first two hours of life and retinopathy during prematurity: a retrospective study from DRYAD
Source: BMC Pediatr. 2025 Mar 8;25:176. doi: 10.1186/s12887-025-05533-8 (PMC11889788; doi:10.1186/s12887-025-05533-8)
Supplement: Supplementary file 1 — Supplementary Material 1 [file 12887_2025_5533_MOESM1_ESM.pdf]

Medical Research Ethics Committee of Affiliated Hospital of Jining Medical University

Approval Document

Approval number	2024C134	Submission date	2024.02.01
Research name	Association between Hematocrit in the First Two Hours of Life and Retinopathy during Prematurity: A Retrospective Study from DRYAD		

Review files:

- ✧ Assignment for technical design
- ✧ Application form for waiving signed consent form
- ✧ Review application form
- ✧ Resume

Statistics comment	Voters	Approval	Modification required prior to approval	Modification required and re-submitted for review	Disapproval	Terminate or suspend its prior approval
	4	4	0	0	0	0

1. Review recommendation:

   None.

2. Review decision:

   ☒Approval, ☐Modification required prior to approval, ☐Modification required and re-submitted for review, ☐Disapproval, ☐Terminate or suspend its prior approval

3. Under continuous review:

   ☐Yes, every ____ month from the date of approval. ☒No.

The ethics committee has the right to change the frequency of continuous review according to the actual situation.

济宁医学院附属医院 医学科研伦理委员会

Medical Research Ethics Committee (seal)

Date: 2024.02.06
